# Supplementary material for: Microwave Irradiation and Glutamic Acid-Assisted Phytotreatment of Tannery and Surgical Industrial Wastewater by Sorghum
Source: Molecules. 2022 Jun 22;27(13):4004. doi: 10.3390/molecules27134004 (PMC9268057; doi:10.3390/molecules27134004)
Supplement: Supplementary file 1 [file molecules-27-04004-s001.zip › Supplementary Figure S1 and S2.pdf]

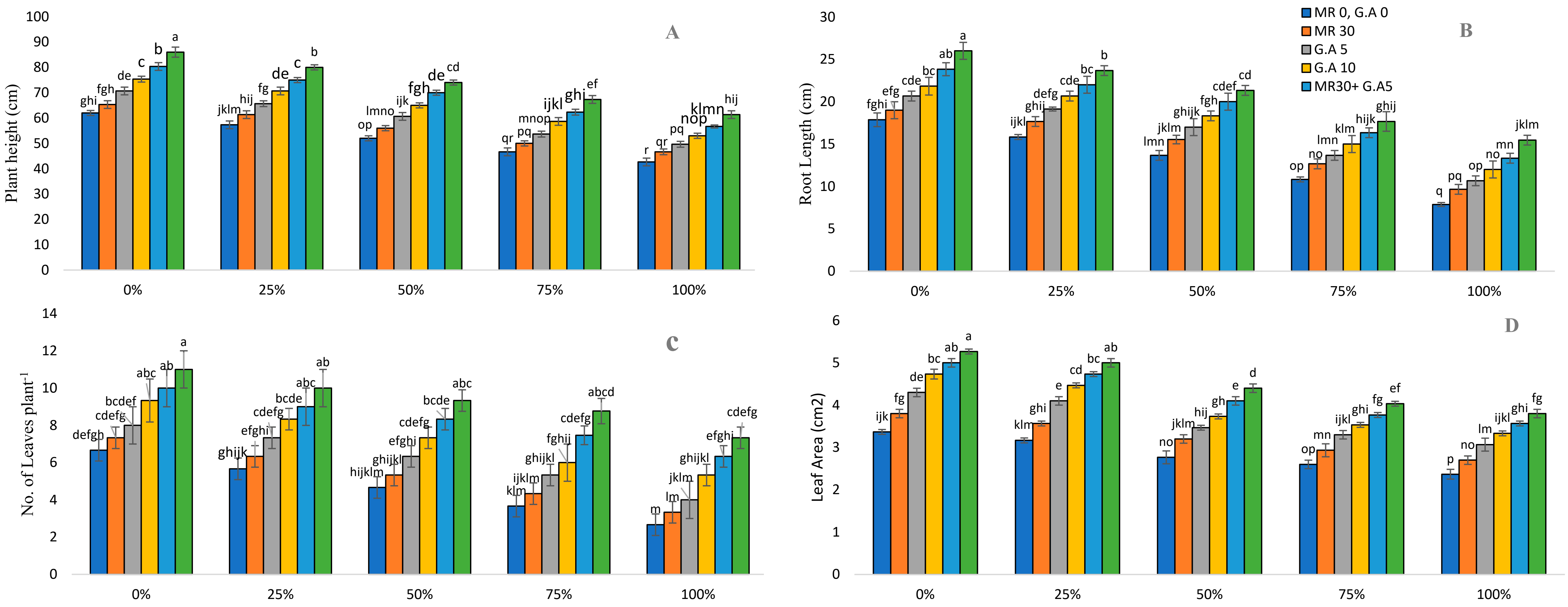

Supplementary Figure S1: Surgical and textile effluents and glutamic acid effects on the plant height (A), root length (B), number of leaves (C) and Leaf area (D) of microwave treated and untreated *Sorghum* L. Values are demonstrated as means of three replicates along with standard deviation. Different normal or italicized small and capital letters indicate that values are significantly different at $P < 0.05$.

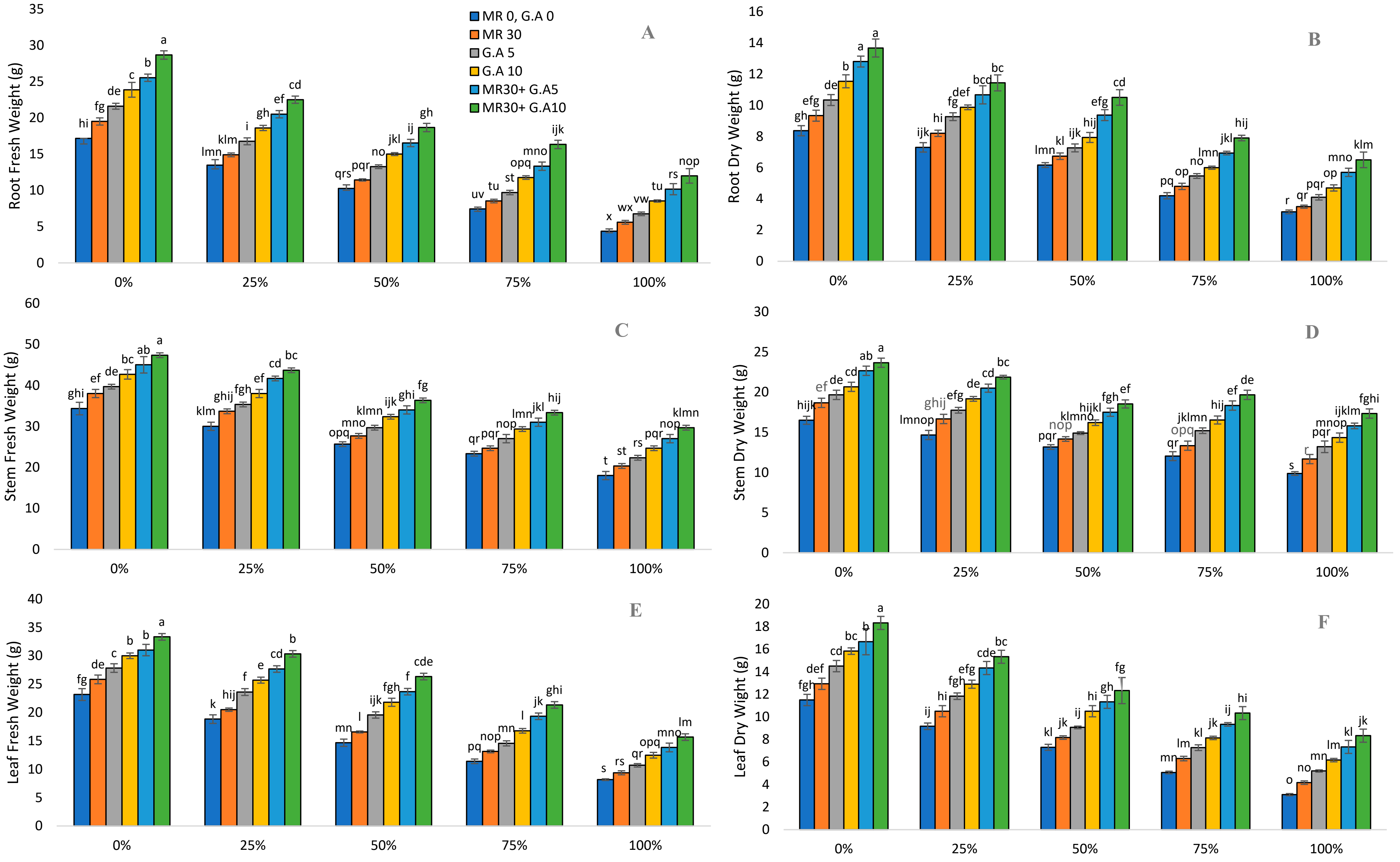

Supplementary Figure S2: Surgical and textile effluents and glutamic acid effects on the root fresh weight (A), root dry weight (B), stem fresh weight (C), stem dry weight (D), leaf fresh weight (E) and leaf dry weight (F) of microwave treated and untreated *Sorghum* L. Values are demonstrated as means of three replicates along with standard deviation. Different normal or italicized small and capital letters indicate that values are significantly different at *P < 0.05*.
