# Supplementary material for: Microwave Irradiation and Glutamic Acid-Assisted Phytotreatment of Tannery and Surgical Industrial Wastewater by Sorghum
Source: Molecules. 2022 Jun 22;27(13):4004. doi: 10.3390/molecules27134004 (PMC9268057; doi:10.3390/molecules27134004)
Supplement: Supplementary file 1 [file molecules-27-04004-s001.zip › Supplementary Table S1 and S2.pdf]

Supplementary Table S1: Experimental Treatments

Treatments	Conc. levels	Treatments	Conc. levels	Treatments	Conc. levels
**T_1**	Ck	T_{11}	WW 25%+ MR 30s+ GA 5mM	T_{21}	WW 75%+ MR 0s +GA 5mM
**T_2**	MR 30s	T_{12}	WW 25%+ MR 30s+ GA 10mM	T_{22}	WW 75%+ MR 0s +GA 10mM
**T_3**	GA 5mM + MR 0s	T_{13}	WW 50% + MR 0s	T_{23}	WW 75%+ MR 30s+ GA 5mM
**T_4**	GA10mM + MR 0s	T_{14}	WW 50%+MR 30s	T_{24}	WW 75%+ MR 30s + G.A 10mM
**T_5**	MR 30s +GA 5mM	T_{15}	WW 50%+ MR 0s +GA 5mM	T_{25}	WW 100% + MR 0s
**T_6**	MR30s+GA 10mM	T_{16}	WW 50%+ MR 0s +GA 10mM	T_{26}	WW+ 100% +MR 30s
**T_7**	WW 25% + MR 0s	T_{17}	WW 50%+ MR 30s+ GA 5mM	T_{27}	WW 100%+ MR 0s +GA 5mM
**T_8**	WW 25% +MR 30s	T_{18}	WW 50%+ MR 30s+ GA 10mM	T_{28}	WW 100%+ MR 0s +GA 10mM
**T_9**	WW 25% + MR 0s + GA 5mM	T_{19}	WW 75% + MR 0s	T_{29}	WW 100%+ MR 30s+ G.A 5mM
**T_{10}**	WW 25%+ MR 0s+ GA 10mM	T_{20}	WW 75%+MR 30s	T_{30}	WW 100%+ MR 30s+ GA 10mM

Ck= Control

WW= Wastewater

MR=Microwave Radiation

GA=Glutamic Acid

Supplementary Table S2: Physic-chemical characterization of surgical and textile industry wastewater

**Parameters**	**Surgical wastewater Concentration**	**Textile wastewater Concentration**
Temperature (In Lab)	24.6 °C	27.4°C
PH value	8.0	7.55
Conductivity	3100 µS/*cm*	3254 µS/*cm*
Total dissolved solids (TDS)	1727 mg/L	2155.0 mg/L
Suplphate	69 mg/L	90 mg/L
Flouride	11.4 mg/L	13.4 mg/L
Total Chlorine	1.6 mg/L	1.9 mg/L
Cyanide	ND	ND
Total Hardness (As $CaCO_3$)	364 mg/L	387 mg/L
Chloride	308 mg/L	360 mg/L
Sulfide	1.3 mg/L	1.2 mg/L
Oil and Grease	8.23 mg/L	10.3 mg/L
Chemical Oxygen Demand (COD)	141 mg/L	145 mg/L
Biochemical Oxygen Demand (BOD)	132 mg/L	137 mg/L
Phenol Compounds	0.2 mg/L	0.2 mg/L
Total Suspended Solids	8132 mg/L	8013 mg/L
Lead (Pb)	1.9 mg/L	2.6 mg/L
Copper (Cu)	0.9 mg/L	1.9 mg/L
Chromium (Cr)	1.1 mg/L	1.6 mg/L
Cadmium (Cd)	2.2 mg/L	2.9 mg/L
Arsenic (As)	1.1 mg/L	1.1 mg/L
Nickel (Ni)	2.4 mg/L	1.9 mg/L
Iron (Fe)	1.28 mg/L	1.0 mg/L
Zinc (Zn)	0.12 mg/L	0.18 mg/L
Manganese (Mn)	0.9 mg/L	0.10 mg/L
Mercury (Hg)	0.9 mg/L	0.13 mg/L
Boron (B)	0.1 mg/L	0.1 mg/L
Selenium (Se)	ND	ND
Chlorine (Cl)	ND	ND
Ammonia (NH_3)	ND	ND
